# African leaders’ views on critical human resource issues for the implementation of family medicine in Africa

**DOI:** 10.1186/1478-4491-12-2

**Published:** 2014-01-17

**Authors:** Shabir Moosa, Raymond Downing, Akye Essuman, Stephen Pentz, Stephen Reid, Robert Mash

**Affiliations:** 1 Department of Family Medicine, University of Witwatersrand, Johannesburg, South Africa; 2 Department of Family Medicine, Moi University, Eldoret, Kenya; 3 Family Medicine Unit, Department of Community Health, University of Ghana, Accra, Ghana; 4 PHC Directorate, University of Cape Town, Cape Town, South Africa; 5 Division of Family Medicine and Primary Care, Stellenbosch University, Stellenbosch, South Africa

**Keywords:** Family medicine, Primary health care, Africa, Development, Family physician, Human resources, Policy

## Abstract

**Background:**

The World Health Organisation has advocated for comprehensive primary care teams, which include family physicians. However, despite (or because of) severe doctor shortages in Africa, there is insufficient clarity on the role of the family physician in the primary health care team. Instead there is a trend towards task shifting without thought for teamwork, which runs the risk of dangerous oversimplification. It is not clear how African leaders understand the challenges of implementing family medicine, especially in human resource terms. This study, therefore, sought to explore the views of academic and government leaders on critical human resource issues for implementation of family medicine in Africa.

**Method:**

In this qualitative study, key academic and government leaders were purposively selected from sixteen African countries. In-depth interviews were conducted using an interview guide. All interviews were audio-recorded, transcribed and thematically analysed.

**Results:**

There were 27 interviews conducted with 16 government and 11 academic leaders in nine Sub-Saharan African countries: Botswana, Democratic Republic of Congo, Ghana, Kenya, Malawi, Nigeria, Rwanda, South Africa and Uganda. Respondents spoke about: educating doctors in family medicine suited to Africa, including procedural skills and holistic care, to address the difficulty of recruiting and retaining doctors in rural and underserved areas; planning for primary health care teams, including family physicians; new supervisory models in primary health care; and general human resource management issues.

**Conclusions:**

Important milestones in African health care fail to specifically address the human resource issues of integrated primary health care teamwork that includes family physicians. Leaders interviewed in this study, however, proposed organising the district health system with a strong embrace of family medicine in Africa, especially with regard to providing clinical leadership in team-based primary health care. Whilst these leaders focussed positively on entry and workforce issues, in terms of the 2006 World Health Report on human resources for health, they did not substantially address retention of family physicians. Family physicians need to respond to the challenge by respondents to articulate human resource policies appropriate to Africa, including the organisational development of the primary health care team with more sophisticated skills and teamwork.

## Introduction

Africa is expected to be a land of opportunity by 2060 [1]. However, extreme poverty, a high burden of disease and poor management of inadequate health resources challenge its growing population. Establishing integrated primary health care is a priority [2,3].

Healthcare in Africa is defined by its human resource (HR) challenge, with large shortages of nurses and more so of doctors, particularly in rural and remote areas [2,4,5]. The overwhelming trend in Africa is towards task shifting in order to reduce dependency on doctors [6]. The 2008 World Health Report on primary health care (PHC) asserts that PHC is dangerously oversimplified with verticalised task-shifting and stresses the need for more sophisticated PHC skills and teamwork [7] based on international evidence of family physicians’ value [8,9]. Unfortunately, the 2006 World Health Report on human resources and subsequent African reviews of PHC do not explore the role of family physicians as part of the primary health care team [2,7]. The World Health Assembly resolved in 2009 that PHC teamwork should include family physicians [10], but important African World Health Organisation (WHO) milestones fail to address the role of family physicians in African primary health care [11-13]. Doctors feel they are expected to fill gaps at rural district hospitals while inadequately equipped nurses and clinical officers drive PHC service delivery [14]. An unclear role for doctors, due to doctor shortages, creates conflict and sets up a vicious cycle of further doctor shortage, as they avoid working in primary care [15]. There is a lack of understanding of family medicine by stakeholders, and as a result, there is limited HR policy support [16]. As family medicine tries to improve health systems globally it is acutely aware of the need to address issues in Africa [17].

As family physicians engage with leaders in Sub-Saharan Africa, little is known of leaders’ views on the implementation of family medicine, and related human resource issues [6]. The purpose of this study was to understand critical human resource issues, including training, to establish family medicine in Africa. This study is part of a larger study which focused on the understanding of family medicine, its implementation challenges and critical human resource challenges.

## Method

### Study design

In-depth interviews were carried out of leaders from countries in Southern, Eastern and Western Africa. Departments of Family Medicine in the PRIMAFAMED Africa Network from sixteen African countries were asked to purposively identify four key country leaders in their country: two in a government department of health and two in an academic institution, who they saw as influential to the implementation of family medicine in the public service. Those leaders with postgraduate education in family medicine and in the private sector were excluded.

### Data collection

The authors, as well as members in the Departments of Family Medicine in the PRIMAFAMED Africa Network, conducted the interviews. Interviewers were all briefed and trained in qualitative interviewing. Interviewers conducted a 30 to 60 minute interview following written informed consent. A standard operating procedure was used to direct the process.

The following open-ended questions were used to explore leaders’ views:

1. Can you tell us about family medicine?

2. What are your thoughts on the role of family medicine in your country?

3. What do you think are the issues in implementing the discipline of family medicine?

4. What are critical human resource issues to establishing family medicine?

5. Do you have anything else to add?

All interviews were conducted in English, except in the Democratic Republic of Congo where interviews were conducted in French and translated into English by the interviewer. All interviews were digitally recorded. The interviewers transcribed their digital recordings *verbatim*. The transcriptions, and interviewing approach, were validated against the digital recordings. Some of the interviewees were re-interviewed for clarity.

### Data analysis

Qualitative data analysis followed the framework method [18]. The authors collectively developed a thematic index with initial transcripts. SP systematically coded all transcripts according to the thematic index, using NVivo9 (QSR International, http://www.qsrinternational.com). SM developed the first set of results from this coding using the framework of the WHO Report 2006 on human resource issues [19]. These results were iteratively completed by Email with inputs from all authors. The overall process was reviewed against the COREQ checklist [20].

### Ethical approval

Ethical approval was given by the University of the Witwatersrand’s Human Research Ethics Committee (Medical) (M110105) in May 2011, Moi University’s Institutional Research Ethics Committee (IREC/2011/78) and the Ethical and Protocol Review Committee of the University of Ghana Medical School (MS-Et/M.4-P5.5/2011-12). There were no monetary rewards provided to respondents. The data produced in the project is confidential, and interviewees are anonymous in all transcripts and analyses. All data, including recordings and transcripts, are to be destroyed after five years as per ethical approval.

## Results

A total of 27 interviews were conducted in nine African countries in 2011 (see Table 1). All respondents held senior posts: heads of department, directors, deputy directors in national or provincial government ministry of health; and vice-chancellor, dean, vice-dean, principal in medical schools and college president in academia. No more details can be made available in order to maintain anonymity.

**Table 1:** Number of respondents per country and sector

**Countries**	**Government**	**Academic**
Botswana	1	0
Democratic Republic of Congo (DRC)	3	1
Ghana	2	2
Kenya	2	2
Malawi	1	2
Nigeria	1	0
Rwanda	2	2
South Africa (SA)	3	1
Uganda	1	1
TOTAL	16	11

Respondents are labelled by country, as either (G)overnment or (A)cademic and by their interview number.

Responses to critical human resource issues in establishing family medicine mostly concerned a) education, b) planning, c) supervision issues, with a smaller theme of d) general HR management issues. There were no marked differences between academic and government leaders’ views (Table 2).

**Table 2:** Themes and sub-themes

**a) Education**	**b) Planning**
• Train family physicians broadly as clinical healthcare leaders	• Address shortage of human resources (especially rurally) and poor planning
• We need to train more family physicians	• Help to organize the primary health care system
• Train them in rural sites	• Be community- and family-based
• Bridge the gap between undergraduate and postgraduate training	
**c) Supervision**	**d) General HR management (minor)**
• Leaders are required	• Recruitment
• Organisational development strategies	• Compensation
	• Systems supports
	• Career choice
	• Lifelong learning
	• Migration

### a) Education

Many needs were expressed around education and training issues.

#### Train family physicians broadly as clinical healthcare leaders

Medical officers were seen as experienced but less knowledgeable than family physicians. Family physicians, on the other hand, were seen as having more integrated knowledge and skills. Family medicine in Africa is currently seen as strongly contextualized to the Sub-Saharan Africa context. A large emphasis was placed on the four major disciplines of internal medicine, surgery, obstetrics-gynaecology and paediatrics, especially for hospital problems:

Family medicine…is a programme that really tries to produce doctors who are well grounded in the areas of dealing with all the major problems within our population. (Kenya A2)

Family medicine could not be exactly as it is in America or in Europe, but it has to integrate local reality…family physician in DRC have to be trained in internal medicine, paediatrics, surgery, obstetrics and gynecology. (DRC A1)

Whilst the family medicine curriculum was viewed as adequate, it was felt that the training needs support, with practical exposure to basic surgery and anaesthetics. There was concern about clinical training outcomes and confidence with procedural skills. However, family physicians were expected to know their limits:

They shouldn’t turn them into super specialists but they should be able to deal with these elements…able to deal with geriatric issues, simple fractures and then refer. (Botswana A1)

Family physicians were viewed as having a broader generalist perspective than other specialists, including a psychosocial approach to issues. Postgraduate training was seen as giving family physicians the ability to think more broadly and to solve problems. Family physicians were expected to have strong health systems management, research and interpersonal skills:

Perhaps the non-technical side…one is also expected to have some basic knowledge in all those other fields because at your level you are a manager. (Kenya G4)

Family physicians were expected to supervise and train PHC teams, and act as leaders in clinical governance. Some interviewees saw new graduates as role models in team leadership, and attributed this to their training:

I understand people are being trained to play that role as being a mentor and supporter of people around clinical governance. (SA G2)

#### We need to train more family physicians

Leaders wanted to increase trainers and training sites. Some felt training was weak, with insufficient trainers. South African leaders expressed a preference for the newer full-time clinical training in registrar posts, compared to the previous distance-based academic qualification. All interviewees wanted to see greater impact:

We need to train more; what we have now is the limitation in numbers. (Ghana G3)

People who were specializing under the older family medicine, and they were doing distance learning. I didn’t think that that worked so well. (SA G2)

#### Train them in rural sites

The discussion centred on locating family physicians in rural areas. There was a view that family physicians would be pivotal in developing training at rural district hospitals, although this might be limited by trainer availability; hence the need for training at central hospitals. South African universities were viewed as models:

I think family physicians can play that role, and playing a pivotal role in the districts and even in establishing satellites for medical schools and, may I say, even for faculties of health sciences. (SA A1)

Regional hospitals were viewed as potential training sites, with rotations between disciplines. It was hoped that the limited number of family physicians available would integrate these to create optimal postgraduate training in family medicine. It was felt that family medicine training in rural areas would lead to better retention of family physicians in rural areas post-training:

The family medicine specialist will then be able bring the people (registrars) starting to say that you’ve been to different areas (clinical departments) but this is actually putting them together (in family medicine time)…if we have this sandwich model. (Ghana G4)

If we make a mistake of training them in big towns then we will produce people who cannot fit in the rural areas where we want most of these people to be. (Kenya G3)

#### Bridge the gap between undergraduate and postgraduate training

Respondents felt that undergraduate training failed to produce doctors suited to PHC. It was felt that current detailed and outdated medical undergraduate training, led by ‘old die-hards’ was more designed to create specialists and should be re-arranged into alternative education models, as experienced in some countries. They felt that undergraduate medical students needed to see family medicine as a discipline on its own:

As they leave medical school, they have not learned to put together the knowledge and skills appreciably. (Kenya A1)

I also see family physicians (in Africa) within potentiality…The curriculum (in Canada) was under the governance or guidance of a family physician so the students were trained as family physicians from the start. (SA G3)

Respondents saw family medicine helping to develop team-based and community-based education and research across the professions, starting at an undergraduate level:

I think that family medicine has got a benefit to bridge that gap…to actually establish that concept of a team approach and working together as a team. (SA A1)

### b) Planning

There was also a broad response on health system planning.

#### Address shortage of human resources (especially rurally) and poor planning

A strong theme was the general human resource shortage, including the lack of trained family physicians. Respondents wanted to increase posts and the output of family physicians quickly. In general, planning was considered poor with regard to HR policy for PHC:

I would have wished to grow the specialty a little faster than we have grown. (Kenya A1)

We’ve been talking a lot about family medicine…but we haven’t had a detailed human resource plan to support this. (SA A1)

The underlying need for trained family physicians was in rural areas. Family physicians were seen as ideal for providing better access to quality care in rural district hospitals but there was poor planning for rural recruitment and retention.

Currently family medicine in Kenya is best suited (for access) because (the) majority of Kenyans still live in peri-urban, rural and hard to reach areas. (Kenya G4)

Family medicine practice require(s) a high level of integrity and a lot of commitment because …very few would want to go and work under, because it is about primary health care. (Kenya G4)

#### Help to organise primary health care system

Respondents felt that the district health system needed to be adapted to integrate family physicians into primary care teams to support nurses and clinical officers (as mid-level workers), supervise medical officers and link up with specialists:

I see family medicine practitioners should be widely available in all districts and providing PHC closer to the people and also working with the lower level health providers, like clinical officers, like the nurses in those localities, and other health providers, nutritionist and so on. (Uganda A1)

Some respondents saw family physicians as administrative ‘heads’, while others saw them as consultants at sub-district level, organizing doctors and all services, to serve the catchment population of health centres. It was felt that family physicians could reduce referrals, and bring care closer to communities:

It is our saviour for developing countries like in Ghana…we feel that if we get the family health practitioners in all the districts then they will be looking down to all the health centers, nurse, doctors…everybody would come under their supervision…if it becomes an octopus, the training will go down to the basics, for people (family physicians) to see what the people (junior healthcare workers) are doing and influence it. (Ghana G4)

These people are team leaders for the district as a whole. (Malawi G3)

It was felt that family physicians understand districts and programmes better than medical officers, including the ability to set standards with clinical governance, and conduct outreach visits and training:

Family medicine, they also have a better understanding on the whole set up, lets say at a district level, in terms of the different programs that are run. (Malawi A1)

#### Be community-based and family-based

In addition to clinical skills, family physicians were expected to be orientated toward the family and community context and to think about a defined population, and not just the patient consulting them:

Another benefit of becoming a family physician is enabling him to be able to interact with community and be able to undertake community diagnosis. (Kenya G4)

### c) Supervision

Many leaders expected family physicians to play a strong supervisory leadership role.

#### Leaders required

Family medicine was seen as pivotal to the supervision, standard setting and organization of the primary health care team. The team was expected to be a more ‘cost-effective production unit’ with a family physician leading a team of different level clinicians including medical officers, clinical officers and nurses:

A family medicine doctor when he goes to the health centre, it will not be only to work, (but) also like a supervisor, a trainer, a campaigner. (Rwanda A1)

There were concerns about confused chains of command, overlapping roles, poor resources and as a result, family physicians having a low impact. Furthermore, family physicians were expected to lead with sensitivity. Whilst there was disquiet about family physicians simply sitting in district offices and acting as administrators, respondents felt that family physicians also need to delegate effectively, and avoid taking on too many tasks with a risk of failure:

You’ve got to feed your way into the system and learn from doing, not sort of being the new kid on the block and that’s my way or the highway kind of thing…otherwise you are going to lose friends and not influence people very quickly. (SA G2)

#### Organisational development strategies

South African respondents suggested strategies: family physicians with different levels of seniority, depending on experience and skills, would work in conjunction with specialists and non-specialist medical officers according to norms related to area, patient needs, district hospital workloads and population profiles. Family physicians were advised to develop these:

At an administrative level, I think there should be a district family physician that is part of the district management team…responsible for all the policy and administrative aspects of implementation even at a clinical level. But I think at an operational level…all those people operationally should report to the head of the entities in which they work. It shouldn’t be difficult. (SA G4)

The danger was felt that if family medicine grows too quickly, family physicians’ would be burdened with unnecessary administrative responsibilities. Family medicine practice also needed local variation where, for example, a family physician may fill a gap in rural areas when there are no anaesthetists but not in urban areas where anaesthetists may be available. It was felt that family physicians (given their skills) should be the lead district specialist if there were other specialists in the districts, as family physicians are able to bring the different specialties together in clinical governance:

If I could design the model, I would take these other specialists out of the equation, (by) sort of having different levels of family medicine…I think that’s something that the discipline needs to think about and come up with some answers. (SA G2)

### d) General human resource management issues

A number of general human resource management issues were raised.

#### Recruitment

It was felt that many doctors do not know about family medicine. Offering scholarships, increasing marketing and developing clearer career paths could change this:

Their need(s) to be a little more initiative, particularly to expose the students to family medicine, like we are exposing the interns. (Kenya A1)

Respondents felt that there were few visible, locally trained family physicians, with a notable lack of professors in family medicine. Whilst foreign support and faculty was welcomed, and seen as valuable for meeting international standards, the respondents were wary of foreign ‘agendas’ and dependencies:

We need professors in family medicine in the country. (DRC G3)

Threats are there, the international things they can bring in their own agendas. (Malawi A1)

### Compensation

It was felt that remuneration and benefits were a big challenge and needed to be brought to the same level as other specialists, especially within the private sector. It was felt that governments need to address motivations to live in remote areas and work in rural district hospitals:

I think people have a perception that family medicine does not attract as good a pay or as good an income as it does for several other specialists…particularly in the private sector. (Uganda A1)

Doctors don’t want to live in places where they are not satisfied, like in remote areas. (Rwanda G2)

### Systems supports

National health insurance was seen as an opportunity for family medicine to reduce specialist work and unduly high private costs:

If you’re looking 20, 30 or 40 years down the line, I suppose that’s the model to aim for…I certainly think that there should be more generalists trained than specialists for South Africa’s needs. (SA G2)

Some sort of insurance system…will bring more patients from private practice into government institutions. Yea? And therefore that will require a different kind of specialist to begin with. (Kenya 1A)

### Career choice

There was a feeling that positions similar to other specialists would also serve to engender respect and attract and retain doctors in family medicine. Family medicine currently holds a ‘second grade’ status. This is partly because there are few trainers and visible role models, and because doctors don’t believe it is professionally and financially satisfying:

In government service you would not be promoted unless you had a specialty. (Kenya A1)

### Lifelong learning

There was a felt need for family physicians, given their wide scope of service, to work in groups to mentor each other:

The ministry will support…posting these physicians, family physicians in places where they can work, ah, they can work together, perhaps in pairs, or in threes. (Kenya A1)

### Migration

Losing well-trained, marketable family physicians to the non-governmental organisation (NGO) sector and to other countries was seen as a threat:

You know somebody talked of not brain drainage but also (of) brain circulation…in the region. (Malawi A1)

## Discussion

In summary, the key themes are: educate doctors in family medicine suited to Africa with procedural skills and holistic care to address the difficulty of recruiting and retaining doctors in rural and underserved areas; plan for primary health care teams, including family physicians; develop new supervisory models in primary health care; and address general human resource management issues.

The WHO argues for people-centred primary care globally, that is based on family medicine principles [7] and for PHC teamwork to include family physicians [10]. There is strong appreciation by the WHO of the potential role family physicians play globally [17]. However, there appears little reflection on this in African PHC [3]. In contrast to this, the viewpoints of leaders in Sub-Saharan Africa are congruent with a growing consensus on a valuable role for family physicians in Africa [14,16,21,22]. Interviewees appreciated the value of family physicians: as improving quality of clinical care, improving the early management of health problems, and providing more appropriate care while reducing inappropriate referrals, as known in the literature [8]. Moreover, they had far-reaching ideas on family physicians’ role in African PHC: filling in the skills and leadership gaps felt in the district health service, especially in rural hospitals; being central to the organisation of PHC; and strengthening clinical supervision with new organisational development strategies. Education was viewed as key to achieving these. Universal health coverage (UHC) featured also. This is consistent with UHC as a growing international priority post the Millennium Development Goals (MDGs) [23].

There is a strong need to review recent African human resource for health efforts to specifically highlight the human resource issues of primary health care [11-13]. The 2006 World Health Report on human resources stressed the need for the right mix in terms of numbers, diversity and competencies for primary health care teams [19]. With regard to this mix, interviewees proposed organising the district health system with a strong emphasis on family medicine in Africa, especially with regard to providing clinical leadership in primary health care teams. Respondents suggested organisational strategies and challenged family physicians to produce HR policy proposals.

A key question will be what the role of family physicians will be in a post-MDG environment where service delivery reform is required with emerging universal coverage systems in Africa. HR policy proposals need to clarify the competencies, structure and limits of primary health care teams, including the role and number of family physicians. This must achieve a cost-effective balance between access and quality in a primary health care system, re-organised for personalised, team-based and community-based care accountable to defined populations [7]. The role of family physicians should include hospital care but should not be defined by it.

It is critical that family medicine education responds to these issues, including championing reform in undergraduate health training and accelerated postgraduate training models to achieve impact. Making an impact at scale requires comprehensive human resource planning for primary health care building on existing WHO frameworks, African country-level efforts and family medicine proposals [19,24-28].

Whilst this positive view by leaders may be flattering to family physicians, it will take more for family medicine to be able to go to scale in Africa. The retention of highly trained health professionals, including family physicians, in rural areas requires a comprehensive approach [6]. Exit human resource issues (recruitment, systems supports, lifelong learning, career choice and migration), as per the discourse of the WHO [19], were rarely raised by these leaders. When these issues were raised, it was mostly about what these leaders expected family physicians to provide for the PHC team. Little was mentioned about supporting family physicians themselves.

This study is limited with the use of multiple interviewers despite fairly uniform adherence to the question guide. Interviewers may also have accessed supporters of family medicine more easily, which could have led to a bias towards supportive views. Additional viewpoints might have emerged if additional countries had been included in the study. The findings cannot be generalised to Africa as a whole. Despite these limitations, the richness of data offers transferability to other contexts and is an important contribution to the development of family medicine in an African context.

## Conclusions

Leaders in Africa identified critical human resource issues in establishing family medicine: improved education, planning, and supervision. Family physicians need to explore deficits in primary health care broadly and to articulate comprehensive and appropriate HR policy proposals to implement family medicine, and strengthen primary health care effectively in Africa. Family physicians also need to manage the high expectations, specialist mind-set and hospital focus in Africa. Cost-effective human resource models in primary health care, with the right staff-mix and skills-mix to enable adequate wide access and quality to defined populations, will be important in the light of UHC in the post-MDG era.

## Abbreviations

HR: human resources; MDG: millennium development goals; NGO: non-governmental organisation; PHC: primary health care; UHC: universal health coverage; WHO: World Health Organisation.

## Competing interests

This research was in the framework of the HURAPRIM (Human Resources in African Primary Health Care) Project, funded by the European Union’s Seventh Framework Programme (FP7-AFRICA-2010) [Grant Agreement number 265727]. The authors declare that they have no other financial or non-financial competing interests.

## Authors’ contributions

SM and RM conceived the study. All authors helped in designing the study, drafting the protocol and interviewing. SP analysed the data, supervised by SM. All authors developed the thematic index, and finalised themes and findings. The authors collectively interpreted this and published an article around understanding of family medicine. Human resource issues, especially training, were seen as a key other finding. SM analysed the data specifically on human resources and presented these by Email to the authors as draft results and draft articles. All authors read and approved the final manuscript.

## Authors’ information

SM, MBChB, MFamMed, MBA, is a senior clinical lecturer in the Department of Family Medicine and the lead researcher in the HURAPRIM Project at the University of the Witwatersrand, South Africa.

SP, MSocSci (Social Anthropology), is a co-researcher in the HURAPRIM Project at the University of the Witwatersrand, South Africa.

RM, MBChB, MRCGP, DRCOG, DCH, FCFP, PhD, is Head of the Division of Family Medicine in Stellenbosch University, South Africa.

SR, BSc Med, MBChB, MFamMed, PhD, is the Glaxo-Wellcome Chair of the PHC Directorate in the University of Cape Town, South Africa.

RD, MD, DTM&H, is lecturer in the Department of Family Medicine in Moi University, Eldoret, Kenya.

AE, BSc, MBChB, FGCP, is the Acting Head of the Family Medicine Unit, Department of Community Health in the University of Ghana, Ghana.
